# Identification of novel genetic loci for osteoporosis and/or rheumatoid arthritis using cFDR approach

**DOI:** 10.1371/journal.pone.0183842

**Published:** 2017-08-30

**Authors:** Rou Zhou, Xu Lin, Ding-You Li, Xia-Fang Wang, Jonathan Greenbaum, Yuan-Cheng Chen, Chun-Ping Zeng, Jun-Min Lu, Zeng-Xing Ao, Lin-Ping Peng, Xiao Chun Bai, Jie Shen, Hong-Wen Deng

**Affiliations:** 1 Department of Endocrinology and Metabolism, The Third Affiliated Hospital of Southern Medical University, Guangzhou, PR China; 2 Department of Gastroenterology, Children’s Mercy Kansas City, University of Missouri Kansas City School of Medicine, Kansas City, MO, United States of America; 3 Center for Bioinformatics and Genomics, Department of Biostatistics and Bioinformatics, Tulane University, New Orleans, LA, United States of America; New Jersey Institute of Technology, UNITED STATES

## Abstract

There are co-morbidity between osteoporosis (OP) and rheumatoid arthritis (RA). Some genetic risk factors have been identified for these two phenotypes respectively in previous research; however, they accounted for only a small portion of the underlying total genetic variances. Here, we sought to identify additional common genetic loci associated with OP and/or RA. The conditional false discovery rate (cFDR) approach allows detection of additional genetic factors (those respective ones as well as common pleiotropic ones) for the two associated phenotypes. We collected and analyzed summary statistics provided by large, multi-center GWAS studies of FNK (femoral neck) BMD (a major risk factor for osteoporosis) (n = 53,236) and RA (n = 80,799). The conditional quantile-quantile (Q-Q) plots can assess the enrichment of SNPs related to FNK BMD and RA, respectively. Furthermore, we identified shared loci between FNK BMD and RA using conjunction cFDR (ccFDR). We found strong enrichment of p-values in FNK BMD when conditional Q-Q was done on RA and *vice versa*. We identified 30 novel OP-RA associated pleiotropic loci that have not been reported in previous OP or RA GWAS, 18 of which located in the MHC (major histocompatibility complex) region previously reported to play an important role in immune system and bone health. We identified some specific novel polygenic factors for OP and RA respectively, and identified 30 novel OP-RA associated pleiotropic loci. These discovery findings may offer novel pathobiological insights, and suggest new targets and pathways for drug development in OP and RA patients.

## Introduction

Osteoporosis (OP) is a major public health problem, affecting millions of people worldwide, particularly postmenopausal women. In the United States, the prevalence of OP is estimated to be greater than 14 million people in 2020 [1]. In China, the population with OP is expected to reach 212 million people by the year 2050 [2]. OP is characterized by low bone mass and micro-architectural deterioration of bone tissue, leading to increased bone fragility and increased susceptibility to fracture [3]. Bone mineral density (BMD) measurement is currently the standard major method for the diagnosis OP and fracture prediction. OP is a polygenetic disorder with a BMD heritability estimated between 50% and 80% [4].

Rheumatoid arthritis (RA) is an autoimmune inflammatory disease affecting approximately 0.5–1% of the world population. RA is characterized by symmetric joint swelling, tenderness and destruction of synovial joints, eventually leading to severe joint destruction and disability [5]. In US, RA affects 1.3 million adults [6]. In China, it is estimated that 0.32%-0.36% people suffer from RA [7]. It is widely recognized that RA is closely associated with both genetic and environmental factors. Twin studies showed that the heritability of RA is about 60% [8].

RA is frequently associated with OP, which may occur in 60–80% of RA patients [9]. In RA, bone is a target of inflammation, which leads to systemic OP, periarticular osteopenia and bone erosion, resulting in dramatically increased risks of fractures and related morbidity, mortality, and healthcare costs. Several cross-sectional studies have shown that a lower BMD was associated with increase risk in OP in patients with RA compared to matched controls [10, 11].

OP is a multifactorial condition, especially in patients with RA. Many studies have demonstrated that OP are closely related to proinflammatory cytokines, such as TNF-a, IL-1, IL-6 and IL-17, which have been shown to be vital in bone resorption [12]. Genetic and environmental factors both contribute to RA-associated OP, and genetic background plays an important role. However, genetic risk factors have not been well defined in RA-associated OP. Research for genetic factors has been focused on screening for polymorphisms in inflammatory cytokine genes and OP candidate genes, such as *VDR* gene and *OPG gene*, to identify shared genes between OP and RA. Current findings accounted for only a small portion of the underlying total genetic variances for OP and RA. In addition, reported results from different studies were largely in disagreement [13–16]. Hence, it is necessary to systematically identify additional common genetic loci associated with OP and RA using a different and new approach.

The conditional false discovery rate (cFDR) is a method recently developed by Andreassen et al. [17], and is powerful for genome-wide association studies (GWAS) analysis using summary statistics. Andreassen et al. has effectively applied and identified previously unsuspected common genetic risk loci for complex diseases, e.g., schizophrenia and cardiovascular disease traits [18], schizophrenia and bipolar disorder [17]. The principle of this method is to test variants for association with the principal phenotype conditional on different strengths of association with a second trait via combining the summary statistics from two different and independent GWAS of two related study traits [17]. We have applied the method and successfully identified some interesting shared genetic risk loci for common complex diseases, e.g., height and FNK BMD [19], Type 2 Diabetes and birth weight [20], Coronary artery disease and BMD [21]. To date, many GWAS for OP and RA have been performed separately and identified multiple genetic risk loci associated with them respectively. The cFDR method can efficiently increase the effective sample size for GWAS analysis of those pleiotropic loci based on existing GWAS data (summary statistics) of two related traits/diseases, hence no need to increase the original GWAS sample sizes with additional sample and data. In this study, we analyzed two large publically accessible GWAS datasets to identify common genetic loci for OP and RA using this new and powerful cFDR method.

## Materials and methods

### GWAS datasets

We acquired two GWAS summary statistics from publically accessible datasets. The first dataset is the association analysis with RA coming from a GWAS meta-analysis of 80,799 subjects (European & Asian) carried out by Okada et al. [22], and we chose European-specific results with 58,284 subjects for this study, to ensure consistency of ethnicity with those used with the following study of OP. This was a meta-analysis that was obtained from 18 studies from Europe to assess European-specific association between RA and more than 8 million genotyped or imputed single-nucleotide polymorphisms (SNPs). The data included summary statistics, which provided the p-values showing association and effect of orientation for each variant after controlling for genomic inflation (due to potential population sub-structure) at both the individual study level and the meta-analysis.

The 2nd dataset is the FNK BMD coming from a GWAS meta-analysis with 33 individual studies, which involved 32,735 European subjects and was performed by the Genetic Factors for OP (GEFOS) Consortium [23]. The dataset provided summary statistics for the associations of the FNK BMD and more than 10 million genotyped and imputed SNPs.

The above two GWAS datasets are independent with no overlapping individuals.

### Data preparation

Firstly, we annotated and mapped SNPs from the two GWAS studies to genes, and combined the summary statistics from the two GWAS studies for the 5,806,739 SNPs that were common in the two studies. Next, in order to generate SNPs being in approximate linkage equilibrium with each other, we applied a linkage disequilibrium (LD) based pruning method in Plink version 19.0 [24]. The pruning process of the dataset used the CEU HapMap 3 genotype and LD information. The pruning algorithm proceeded with a window of 20 SNPs, in which LD between each pair of SNPs was assessed, and if the LD was greater than 0.2 then one of the pair of SNPs was removed. The LD threshold of 0.2 was consistent with other using the cFDR method papers. For the pairs with the LD greater than 0.2, we adopted minor allele frequency (MAF) pruning method and removed the SNP of the smaller MAF. After this initial removal of SNPs, the window shifted 5 SNPs forward, and repeated the procedure until no pairs of SNPs being in high LD. Finally, there were 176,331 SNPs remained to be used in the analysis after pruning.

In order to ensure that the genetic variance estimates from each SNP are not inflated owing to population structure, adjustment of GWAS results with genomic control is necessary. Because the authors from the two original GWAS datasets had previously applied genomic control rigorously, there was no need to apply this adjustment again in this study.

### Statistical analysis

The concept of the cFDR extends from the unconditional false discovery rate (uFDR), which is defined as the probability of a false positive association for a set of variants in the single phenotype case [25]. Similarly, the cFDR applies this idea to the two related phenotype case and is characterized as the probability that a given SNP has a false positive association with the principal phenotype given that the p-values for both the principal and conditional phenotypes are less than or equal to the observed p-values.

$$cFDR\left(p_{i}|p_{j}\right)=\mathrm{Pr}\left(H_{\;0}^{\left(i\right)}|P_{i}\leq p_{i},P_{j}\leq p_{j}\right)$$ (1)

where the observed strength of association that a given SNP is associated with the principal phenotype is expressed as *p*_*i*_, and the observed significance level that the same SNP is associated with the conditional phenotype is expressed as *p*_*j*_. The $H_{\;0}^{\left(i\right)}$ represents the null hypothesis that a given SNP is not associated with the principal trait [19].

According to the steps outlined by Andreassen *et al*. [17], we computed the cFDR for each variant when FNK BMD is the principal phenotype conditioned on strength of association with RA (FNK BMD | RA) as well as the reverse (RA | FNK BMD). In order to confirm if the cFDR method leads to the enrichment of associated SNPs, we consecutively restricted the subset of SNPs that were tested based on the level of significance for the correlation of each variant with the conditional trait applying the following criteria for *P*_*j*_ *≤* *p*_*j*_, such as *P*_*j*_ *≤* *1* (all SNPs), *P*_*j*_ *≤* *0*.*1*, *P*_*j*_ *≤* *0*.*01*, *P*_*j*_ *≤* *0*.*001*. SNPs with the cFDR value smaller than 0.05 were regarded to be significantly related to the principal phenotype.

In order to evaluate the enrichment of association in comparison with that expected under the null hypothesis, we drew conditional quantile-quantile (Q-Q) plots based on changing levels of significance in the conditional trait. The Q-Q plot shows a graph with the observed distribution of p-values plotted versus the expected distribution of p-values under the null. The y-axis represent the nominal p-values (–log_10_(p)) and the x-axis represent the empirical quantiles (–log_10_(q)). The degree of leftward shift from the expected null line intuitively shows pleiotropic enrichment.

Conditional Manhattan plots were used to display the independent loci associated with RA, given their association with FNK BMD as well as the reverse. All the SNPs after pruning within an LD block and their chromosomal location in the genome are shown in these plots. When a–log_10_(p) value is greater than 1.3, the variant was considered to be significantly associated with the principal phenotype considering the association of that variant with the conditional phenotype.

To identify loci that are associated with both FNK BMD and RA, we computed the conjunction cFDR (ccFDR) value after computing the cFDR(FNK BMD | RA) as well as the reverse cFDR(RA | FNK BMD). The ccFDR is characterized as the probability that a given SNP has a false positive association with both the principal and conditional phenotypes. The maximum cFDR value of the two cFDR values is selected as the ccFDR [17].

All SNPs with a ccFDR value smaller than 0.05 were determined to be significantly associated with both traits. In order to show the locations of pleiotropic genetic variants, we presented a conjunction Manhattan plot.

### Functional term enrichment analysis

We performed functional term enrichment analyses using the GO terms database [26] and evaluated the potential functions of the trait associated loci identified by cFDR and pleiotropic loci identified by ccFDR. We then classified trait-associated loci according to their known biological processes and molecular functions. This bioinformatic analysis is intended to partially assess the validity and functional relevance of our findings by identifying gene sets that are significantly related to bone metabolism and/or RA processes.

## Results

### Assessment of pleiotropic enrichment

Conditional Q-Q plots are used to visualize the distribution of p-values and assess the pleiotropic enrichment. The distribution of p-values follows the null distribution diagonal line under the null hypothesis. The enrichment of genetic association would appear to deflect leftwards from the null line because the association of principal phenotype depends on the more stringent association criteria of the conditional phenotype. The larger the interval between the conditions Q-Q plots, the greater the degree of pleiotropic-effect of the genes shared between the two traits.

**Fig 1:**
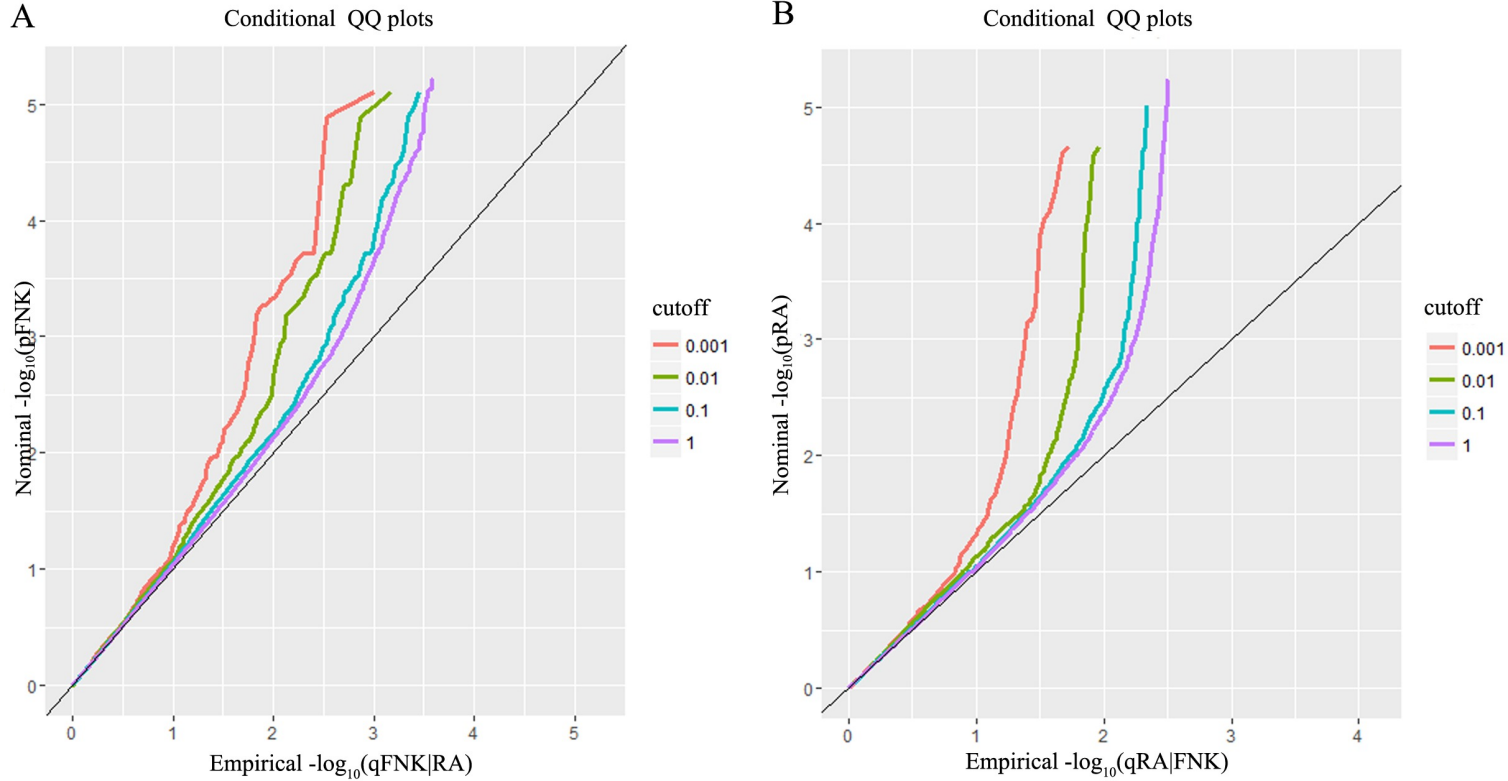
Conditional QQ plots. Conditional QQ plots of nominal versus empirical –log_10_ p-values for (A) FNK BMD as a function of significance of the association with RA, and (B) RA as a function of significance of the association with FNK BMD. The diagonal line represents the null hypothesis.

The conditional Q-Q plot of the FNK BMD giving nominal p-values associated with RA (Fig 1A) reveals some enrichment at the different significance thresholds of RA. The presence of leftward shift indicates that the number of true associations for a given RA p-value increase when the analysis is limited to include SNPs of more significant associations. From the conditional Q-Q plot for RA given FNK BMD (Fig 1B), we can observe a larger separation between the different curves, which indicate that there may be a stronger enrichment for RA (given FNK BMD) than that for FNK BMD (given RA).

### FNK BMD loci identified with cFDR

**Fig 2:**
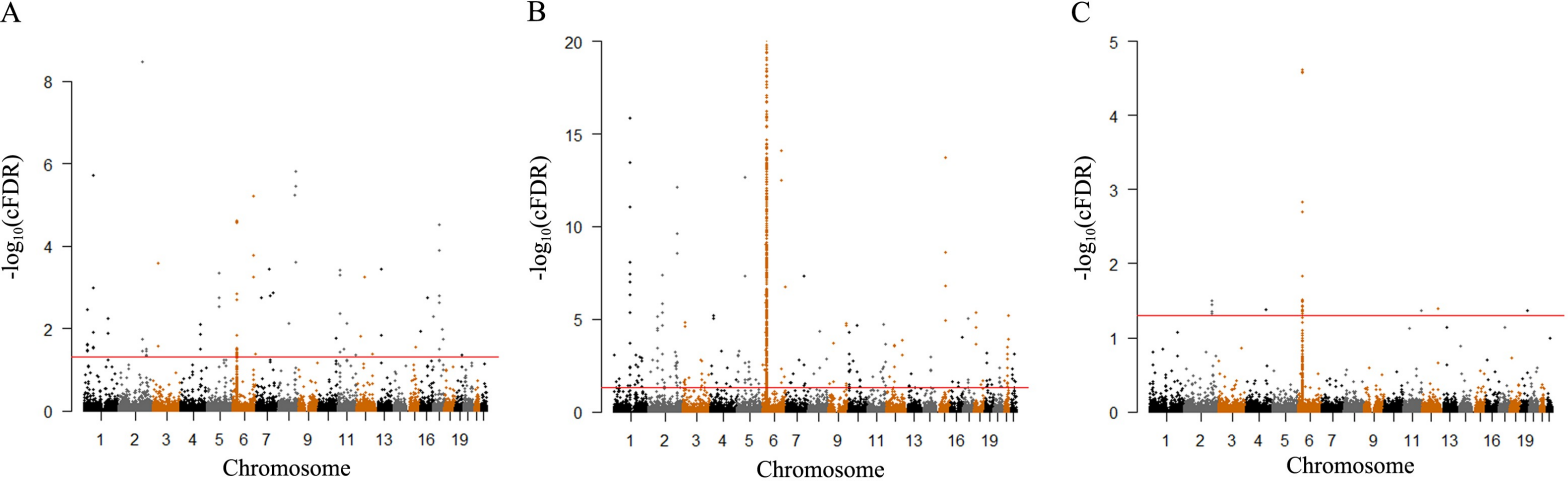
A) ‘‘Conditional Manhattan plot” of conditional –log_10_ FDR values for FNK BMD given RA (FNK BMD|RA); B) “Conditional Manhattan plot” of conditional –log_10_ FDR values for RA given FNK BMD(RA|FNK BMD); C) “Conjunction Manhattan plot” of conjunction –log_10_ FDR values for FNK BMD and RA. SNPs with conditional -log_10_ FDR>1.3 (i.e. FDR<0.05) are shown above the red line.

We identified a total of 88 significant SNPs (cFDR < 0.05) for FNK BMD variation associated with RA (Fig 2A), which were located on 16 different chromosomes. Of the 88 SNPs, 53 had p-values smaller than 1x10^-5^ and 18 reached genome-wide significance at 5x10^-8^ in the original meta-analysis for FNK BMD [23]. There are four SNPs among the eighteen SNPs—rs7554551 [27], rs6710518 [28], rs1038304 [29], rs228768 [30], that were previously reported/identified to be associated with BMD or OP, and the other fourteen were not found reported/identified in earlier studies. Furthermore, these 88 variants identified to be associated with FNK BMD are enriched in several skeletal metabolism related functional terms (Table 1), such as “positive regulation of bone mineralization”, “negative regulation of ossification”, “osteoblast development”, “positive regulation of chondrocyte differentiation”, and “negative regulation of canonical Wnt signaling pathway”.

**Table 1:** Functional term enrichment analysis.

GO Term	Total size of pathway genes	Gene Count	*P*-value	Specific gene names	Benjamini
**FNK BMD loci**					
Positive regulation of bone mineralization	35	3	0.0395	*MEF2C*, *WNT4*, *SMAD3*	0.2881
Negative regulation of ossification	16	2	0.0263	*MEF2C*, *SOST*	0.7617
Osteoblast development	17	2	0.0263	*PTHLH*, *SMAD3*	0.7639
Positive regulation of chondrocyte differentiation	19	2	0.0497	*SMAD3*, *SOX6*	0.7841
Negative regulation of canonical Wnt signaling pathway	163	3	0.0395	*WNT4*, *SOST*, *PARK2*	0.8284
**RA loci**					
Antigen processing and presentation	55	18	1.38E-20	*HLA-DMB*	8.52E-18
Immune response	421	32	4.59E-17	*HLA-DQB2*, *HLA-DMA*, *HLA-DOB*, *ETS1*	1.42E-14
T cell costimulation	78	12	9.89E-10	*HLA-DQB2*, *PTPN11*	1.75E-07
Positive regulation of T cell proliferation	60	7	5.35E-05	*HLA-DMB*	0.0050
Negative regulation of T cell proliferation	37	5	7.38E-04	*IL2RA*, *HLA-DRB1*, *CTLA4*	0.0495
Regulation of immune response	178	10	1.74E-04	*MICA*, *ICAM3*, *NCR3*	0.0143
Negative regulation of tumor necrosis factor production	38	4	8.61E-03	*PTPN22*, *TNFAIP3*, *NFKBIL1*	0.3276
Negative regulation of inflammatory response	79	5	1.17E-02	*IL2RA*, *PTPN2*, *ETS1*, *TNFAIP3*	0.3952
Innate immune response	430	11	2.25E-02	*REL*, *TRIM31*, *C2*,	0.5528

Gene ontology enrichment analysis was carried out using David [26], and “*P*-value<0.05” suggests the term is significantly more enriched than random chance.

### RA gene loci identified with cFDR

We identified a total of 778 significant SNPs (cFDR < 0.05) for RA variation associated with FNK BMD (Fig 2B), which were located on 22 different chromosomes. Of the 778 SNPs identified, 567 had p-values smaller than 1x10^-5^ and 437 reached genome-wide significance at 5x10^-8^ in the original meta-analysis for RA [22].

In order to carry out the functional term enrichment analysis for these results, we chose 633 variants with cFDR < 0.01 for analysis. The results shown a large number of RA loci were enriched in several immune system related functional terms (Table 1), such as “immune response”, “antigen processing and presentation”, “T cell costimulation”, “positive/negative regulation of T cell proliferation”, “negative regulation of tumor necrosis factor production”, “negative regulation of inflammatory response”, “innate immune response”, and “regulation of immune response”.

### Pleiotropic gene loci for both FNK BMD and RA

We applied the ccFDR value to identify genetic loci associated with both FNK BMD and RA. The ccFDR is characterized as the probability that a given SNP has a false positive association with both the principal and conditional phenotypes. Furthermore, we constructed the ccFDR Manhattan plot (Fig 2C) to graphically show the distribution of the pleiotropic loci identified with the two traits. We identified 30 independent pleiotropic loci significantly (ccFDR < 0.05) associated with both traits (Table 2). 18 of the 30 variants were located within the region of the major histocompatibility complex (MHC), which is a genomic region located at 6p21.3 and is essential in the control of the immune system [31], and is previously confirmed to be associated with RA [32] and OP [33]. Of the 18 SNPs, 13 SNPs were located within the class III region of the MHC—rs9378164 (*LY6G5C*), rs1266071 (*BAT5*), rs2734335 (*C2*), rs401775 (*SKIV2L*), rs6909427, rs547261, rs9368716, rs1265762, rs2073046 (*C6orf10*), rs3115576, rs9268055, rs2395114 and rs4713518 (*NOTCH4 and C6orf10*), and other 5 SNPs were located within the class II region of the MHC—rs35571244 and rs35599935 (*PPP1R2P1* and *HLA-DMB*), rs34043227 (*HLA-DQB2* and *HLA-DOB*), rs3101944 (*HLA-DMB* and *HLA-DMA*), and rs1367727 (*HLA-DMA* and *BRD2*). Twelve other variants of the 30 identified pleiotropic variants are located on 6 different chromosomes: seven variants ((rs17016749 (*INPP4B*), rs2880389 (*BOLL* and *PLCL1*), rs1318867 and rs11684176 (*PLCL1*), rs11066320 (*PTPN11*), rs1109241 (*ETS1* and *FLI1*), and rs9469623 (*LOC100132252* and *GRM4*)) are located at or close to the genes that are related to RA and other immune associated traits or OP and other bone related traits [22, 34–38], and five variants ((rs13413470 (*RFTN2*), rs7340470 (*RFTN2* and *MARS2*), rs700653 (*BOLL*), rs10947432 (*C6orf125*), and rs2099102 (*EHD2*)) are novel and not related to any of the two diseases or related traits after a thorough literature search (OMIM and PubMed).

**Table 2:** Conjunction cFDR: Pleiotropic loci in FNK BMD and RA.

SNP	Chr	Role	Neighbor Gene	Raw *P*-Value			cFDR		ccFDR
				FNK	RA		FNK|RA	RA|FNK	
rs13413470	2	intron	*RFTN2*	6.58E-04	5.10E-04		4.39E-02	1.17E-02	4.39E-02
rs7340470	2	NA	*RFTN2|MARS2*	5.83E-04	9.30E-05		3.57E-02	2.39E-03	3.57E-02
rs700653	2	intron	*BOLL*	4.75E-04	2.20E-05		4.76E-02	9.31E-04	4.76E-02
rs2880389	2	NA	*BOLL|PLCL1*	1.92E-04	7.90E-05		3.17E-02	3.22E-03	3.17E-02
rs1318867	2	intron	*PLCL1*	2.26E-04	1.30E-04		3.20E-02	4.60E-03	3.20E-02
rs11684176	2	intron	*PLCL1*	1.91E-04	6.90E-04		4.39E-02	2.79E-02	4.39E-02
rs17016749	4	intron	*INPP4B*	4.74E-05	1.80E-03		1.40E-02	4.19E-02	4.19E-02
rs9378164	6	near-gene-3	*LY6G5C*	2.96E-04	1.20E-08		3.05E-02	6.06E-07	3.05E-02
rs1266071	6	intron	*BAT5*	6.35E-03	1.80E-129		3.18E-02	3.93E-127	3.18E-02
rs2734335	6	near-gene-5	*C2*	5.52E-03	1.90E-122		3.31E-02	4.22E-120	3.31E-02
rs401775	6	intron	*SKIV2L*	6.06E-03	5.30E-89		3.86E-02	9.63E-87	3.86E-02
rs3115576	6	NA	*NOTCH4|C6orf10*	6.14E-03	1.50E-141		3.27E-02	3.67E-139	3.27E-02
rs9268055	6	NA	*NOTCH4|C6orf10*	1.25E-02	1.10E-111		3.67E-02	2.04E-109	3.67E-02
rs2395114	6	NA	*NOTCH4|C6orf10*	1.29E-05	1.00E-250		2.58E-05	1.87E-249	2.58E-05
rs4713518	6	NA	*NOTCH4|C6orf10*	7.95E-06	1.00E-250		2.39E-05	5.10E-249	2.39E-05
rs6909427	6	intron	*C6orf10*	1.10E-02	1.10E-111		3.84E-02	2.27E-109	3.84E-02
rs547261	6	intron	*C6orf10*	1.11E-03	1.00E-250		1.47E-03	1.01E-248	1.47E-03
rs9368716	6	intron	*C6orf10*	3.30E-04	1.30E-167		1.98E-03	6.92E-166	1.98E-03
rs1265762	6	intron	*C6orf10*	2.24E-03	1.10E-117		1.46E-02	1.24E-115	1.46E-02
rs2073046	6	intron	*C6orf10*	1.07E-05	1.00E-250		2.68E-05	2.75E-249	2.68E-05
rs34043227	6	NA	*HLA-DQB2|HLA-DOB*	1.47E-02	2.70E-229		4.11E-02	1.74E-226	4.11E-02
rs35571244	6	NA	*PPP1R2P1|HLA-DMB*	1.14E-02	1.60E-170		4.35E-02	6.76E-168	4.35E-02
rs35599935	6	NA	*PPP1R2P1|HLA-DMB*	1.09E-02	2.40E-180		4.34E-02	1.18E-177	4.34E-02
rs3101944	6	NA	*HLA-DMB|HLA-DMA*	1.22E-02	4.60E-137		4.47E-02	1.39E-134	4.47E-02
rs1367727	6	NA	*HLA-DMA|BRD2*	9.27E-03	1.10E-134		4.50E-02	3.34E-132	4.50E-02
rs10947432	6	intron	*C6orf125*	5.29E-04	3.00E-06		4.69E-02	1.33E-04	4.69E-02
rs9469623	6	NA	*LOC100132252|GRM4*	4.67E-04	7.10E-04		4.34E-02	1.78E-02	4.34E-02
rs1109241	11	NA	*ETS1|FLI1*	4.02E-04	5.20E-05		4.29E-02	2.03E-03	4.29E-02
rs11066320	12	intron	*PTPN11*	5.32E-04	2.50E-05		4.06E-02	8.34E-04	4.06E-02
rs2099102	19	near-gene-5	*EHD2*	3.07E-04	1.40E-03		4.30E-02	3.59E-02	4.30E-02

The following abbreviations are used: chr, chromosome; FNK BMD,Femoral neck bone mineral density; RA, Rheumatoid arthritis; cFDR, conditional false discovery rate; and ccFDR,.conjunction conditional false discovery rate.

## Discussion

In this study, we discovered 88 FNK BMD susceptibility loci, which included 18 loci reached genome-wide significance at 5x10^-8^ in the original meta-analysis for FNK BMD [23]. And we also identified 778 RA susceptibility loci, which included 567 loci reached genome-wide significance at 5x10^-8^ in the original meta-analysis for RA [22]. Most importantly, we reported 30 new OP-RA associated pleiotropic loci that have not been reported in previous OP or RA GWAS. Our study represents the first systematic effort to explore the genetic basis of OP and RA in a large sample set. We identified the pleiotropic effects of variants associated with OP and RA by combining the summary statistics from two different GWAS meta-analyses. Compared with the existing standard single phenotype analysis, synchronous analysis for multiple related traits not only increase discovery of trait-associated variants under original datasets for individual traits, but also provide a new insight to further detect the common genetic mechanisms between associated phenotypes.

We carried out the pleiotropic analysis using a cFDR approach, which was recently developed by Andreassen et al. [17]. The basic idea of cFDR is that variants are more likely to exert a true effect when the variants have significant effects in both traits. In the original simulation of the cFDR approach, the authors demonstrated that cFDR approach resulted in 15–20 times’ increase of the number of non-null SNPs with a local FDR smaller than 0.05, when compared with the uFDR. It’s well known that the traditional meta-analysis method can also increase statistical power, but it can only detect loci that have largely or dominantly the same direction of allelic effects on both traits. In contrast, cFDR method can detect loci regardless of their effect directions [39].

In the following, we highlight some of the salient discoveries from this study for their potential significance to OP and/or RA.

### MHC

Among the 30 SNPs associated with both FNK BMD and RA, 18 were located within MHC region, which is the most vital genomic regions associated with RA. Those 18 SNPs are located at or near the following genes that have been reported to be related to RA: *C6orf10* [40], *NOTCH4* [41], *HLA-DQB2* [42], *HLA-DMB* [43], *HLA-DMA* [43], and *BRD2* [44]. Although we do not find any report about association between these gene and OP or related traits, some other MHC genes have been identified to be associated with OP in previous research [33]. In addition, it was estimated that approximately 40% of the expressed genes in MHC have immune system function [45]. There is increasing evidence of an association between the immune system and bone [46]. The bone remodeling could be influenced by proinflammatory cytokines (eg., IL-1, IL-6, TNF-a, M-CSF, PGE2) primarily through regulating the osteoprotegerin (OPG) / receptor activator of nuclear factor-kB (RANK) / RANK ligand (RANKL) [12].

### PLCL1

The two pleiotropic loci (rs1318867, rs11684176) were located in the intron of *PLCL1* (phospholipase C like 1), an OP candidate gene, which was found to be associated with variation in hip bone size [34]. As for the relationship between *PLCL1* and RA, at present, there is no study reported. However, variants of the *PLCL1* gene had been proven to be associated with other autoimmune diseases, such as Crohn’s disease (CD) [47], systemic lupus erythematosus (SLE) [35]. Clinically, multiple autoimmune diseases (ADs) can be observed in cluster [48], including families with RA, suggesting some degree of common genetic susceptibility.

### PTPN11

The SNP rs11066320 is located in the intronic region of the *PTPN11* (protein tyrosine phosphatase, non-receptor type 11) gene, which encodes Src homology region 2 (SH2)-containing protein tyrosine phosphatase 2 (SHP-2). SHP-2 belongs to the protein tyrosine phosphatase family, which have been involved in regulating intracellular signal transduction initiated via multiple different growth factors and cytokine receptors [49]. A recent GWAS reported that a RA risk locus (dbSNP ID = rs10774624) is related to the *PTPN11* gene, indicating a role for *PTPN11* in RA [22]. Additionally, the *PTPN11* gene is significantly overexpressed in RA fibroblast-like synoviocytes (FLS) compared with osteoarthritis (OA) FLS, suggesting a novel role for SHP-2 in promoting RA FLS invasiveness [50]. On the other hand, SHP-2 acts as a vital role in the RANK/NFATc1 signaling pathway, and regulates osteoclastogenesis and bone mineral homeostasis [51]. Furthermore, SHP-2 has been shown to be important in controlling osteoblast differentiation, proliferation and metabolism [52].

### ETS1

The SNP rs1109241 (11p11.12) is located close to the *ETS1* (ETS proto-oncogene 1, transcription factor) and *FLI1* (Fli-1 proto-oncogene) gene, which have been reported to be associated with RA susceptibility [53]. ETS-1 is part of the ETS family of transcription factors, primarily expressed in lymphoid cells, acts as relevant roles in the lymphocyte development, apoptosis and inflammation and controls the expression of abundant immune-related genes [54]. Several GWAS demonstrated that *ETS1* is associated with RA in Europeans [22], and Asians [7]. Furthermore, Ets-1 is also expressed in pre-osteoblast and osteoblast cells, and studies have revealed that Ets-1 exerts profound effect on osteoblast differentiation and bone development via various mechanisms [55].

### INPP4B

The SNP rs17016749 (4q31.21) is located in the intronic region of the *INPP4B* (inositol polyphos-phate-4-phosphatase type II B) gene, which encodes the inositol polyphosphate 4-phosphatase type II, one of the enzymes involved in phosphatidylinositol signaling pathways [37]. Ferron et al. revealed that *INPP4B* acts as a major regulator of osteoclast differentiation by modulating calcium signaling and NFATc1 pathway. They also demonstrate that *INPP4B* in humans is a novel determinant of OP susceptibility locus via genetic analysis [56].

In summary, our study demonstrated that there is significant pleiotropy between OP and RA by assessing pleiotropic effects in the framework of a conditional analysis. We identified several novel pleiotropic loci for OP and RA. Our results bring further insight into the common genetic influences of OP and RA. Hence, in order to confirm and explore the generality of the results identified in this study, other ethnic populations and animal or cell experiments are needed in future studies.
